# Supplementary material for: Effect of different resumption strategies to flatten the potential COVID-19 outbreaks amid society reopens: a modeling study in China
Source: BMC Public Health. 2021 Mar 29;21:604. doi: 10.1186/s12889-021-10624-z (PMC8006128; doi:10.1186/s12889-021-10624-z)
Supplement: Supplementary file 1 — Additional file 1. [file 12889_2021_10624_MOESM1_ESM.docx]

3 February (890-2345 19381)China's total mask output has topped 10 million pieces daily as domestic mask producers resumed 60 percent of production capacity, said the Ministry of Industry and Information Technology (MIIT).(mask 60%)

<http://english.www.gov.cn/statecouncil/ministries/202002/03/content_WS5e377a15c6d0a585c76ca4c9.html>

10 February (381-2097 37626)China's major courier firms resumed operations. Some companies like SF Express and JD Logistics maintained their delivery services during the holiday. The State Post Bureau last week urged courier companies to restore over 40 percent of their normal handling capacity in the middle of this month and continue to increase their handling capacity according to the development of the epidemic outbreak.

<http://english.www.gov.cn/news/topnews/202002/10/content_WS5e413f6cc6d04ea953b7b870.html>

Over 92 percent of more than 7,000 supermarket outlets in 34 large and medium-sized cities across the country have also resumed operation, Zhu Xiaoliang, director of the ministry's market system development department, told an online news conference

<http://english.www.gov.cn/statecouncil/ministries/202002/10/content_WS5e415ddfc6d04ea953b7b8b2.html>

11 February (377-1638 38800)Enterprises providing essential material support and services for the epidemic control efforts such as medical supplies, energy, logistics and transportation have resumed production as the nation, except hard-hit Hubei province, gradually returns to work this week. About 94.6 percent of the country's major grain production and processing firms have resumed production, as have 57.8 percent of the coal mines, said Cong, adding that the supply of natural gas, electricity and refined oil is sufficient.

<http://english.www.gov.cn/statecouncil/ministries/202002/11/content_WS5e42433cc6d0595e03c20690.html>

12 February (1820-13332 52526)Farmers across China resume production

<http://english.www.gov.cn/news/photos/202002/12/content_WS5e434eafc6d0595e03c207b0.html>

13 February (258-4832 55748)China's mask production capacity utilization rate reaches 94%

<http://english.www.gov.cn/statecouncil/ministries/202002/13/content_WS5e453909c6d0595e03c20a6a.html>

19 February (45-349 56303)China has resumed 70.2 percent of its coal production capacity with output recovering steadily

<http://english.www.gov.cn/statecouncil/ministries/202002/19/content_WS5e4c93d8c6d0595e03c2107c.html>

China's courier sector has resumed over 40 percent of its normal delivery capacity with over 2 million deliverymen racing against the ongoing epidemic.

<http://english.www.gov.cn/news/topnews/202002/19/content_WS5e4ce2ffc6d0595e03c210dd.html>

20 February (258-631 54965)China has gradually resumed construction of major railway projects

<http://english.www.gov.cn/news/topnews/202002/20/content_WS5e4e38c2c6d0595e03c211d6.html>

24 February (9-499 47672)China will speed up the resumption of delivery services to aid the nation's epidemic control and prevention efforts and make sure people can get deliveries of daily necessities amid the ongoing novel coronavirus outbreak. China Post, SF Express and JD have almost resumed full operations, while other major courier companies have resumed 66.7 percent of work capacity.

<http://english.www.gov.cn/statecouncil/ministries/202002/24/content_WS5e530bc5c6d0595e03c214eb.html>

25 February (5-401 45604)Production has been restored at 67.4 percent of steelmakers, with a restoration rate of 86.3 percent at nonferrous metals businesses.

<http://english.www.gov.cn/statecouncil/ministries/202002/25/content_WS5e54717cc6d0595e03c21737.html>

26 February (24-409 43258)Some tourist attractions in low-risk regions of the novel coronavirus outbreak have gradually resumed opening to the public (Culture and Tourism)

<http://english.www.gov.cn/statecouncil/ministries/202002/26/content_WS5e563ad0c6d0c201c2cbd033.html>

27 February (9-318 39919)More than 30 percent of China's small and medium-sized enterprises (SMEs) have resumed work and production. Manufacturing firms had a recovery rate of 43.1 percent. online education and information technology service providers achieved over 40 percent of resumption.

<http://english.www.gov.cn/statecouncil/ministries/202002/27/content_WS5e5786c6c6d0c201c2cbd180.html>

2 March (11-114 30004)The construction of China's major water conservation projects has been resumed. As of the end of February, 80 projects in 22 provincial-level regions had resumed construction, accounting for 72.7 percent of the total.

<http://english.www.gov.cn/statecouncil/ministries/202003/02/content_WS5e5cd282c6d0c201c2cbd5e4.html>

4 March (3-134-2 25352)Around 45 percent of China's small and medium-sized enterprises (SMEs) had resumed work

<http://english.www.gov.cn/statecouncil/ministries/202003/04/content_WS5e5fa99fc6d0c201c2cbd93a.html>

China has resumed 83.4 percent of its coal production capacity amid the fight against the novel coronavirus (COVID-19) outbreak, the National Energy Administration (NEA) said on March 4. As of March 3, all coal mines outside Hubei province, the epicenter of the outbreak, had resumed production

<http://english.www.gov.cn/statecouncil/ministries/202003/04/content_WS5e5fb2aec6d0c201c2cbd95e.html>

5 March (1-126-16 23784)About 92 percent of the 362 key seed enterprises followed by the ministry have resumed production so far, with their production capacity at 62 percent(agricultural material supply)

<http://english.www.gov.cn/statecouncil/ministries/202003/05/content_WS5e60c588c6d0c201c2cbda10.html>

Nursing homes in county-level regions with no new confirmed infections can admit new applicants who have not visited high-risk places, contacted people from such areas or shown symptoms of COVID-19 (Civil Affairs)

<http://english.www.gov.cn/statecouncil/ministries/202003/05/content_WS5e60bce1c6d0c201c2cbda09.html>

6 March (1-74-24 22177)major transportation routes and 78 percent of rural roads linking key producing regions have been connected. the majority of seed or fertilizer manufacturers have resumed production, and 78 percent of retailers surveyed by the ministry have also restarted business

<http://english.www.gov.cn/statecouncil/ministries/202003/06/content_WS5e61a2cfc6d0c201c2cbda48.html>

China's courier sector has resumed over 80 percent of its normal delivery capacity with about 3 million deliverymen racing against the ongoing epidemic, according to the State Post Bureau (SPB).

<http://english.www.gov.cn/statecouncil/ministries/202003/06/content_WS5e61e3fcc6d0c201c2cbda9d.html>

12 March (0-5-3 13526)Up to 2,836 entry and exit authorities at or above the county level in China have resumed service, accounting for 87 percent of the total 3,250 such authorities, according to the National Immigration Administration (NIA).

<http://english.www.gov.cn/statecouncil/ministries/202003/12/content_WS5e6a2b02c6d0c201c2cbe2bc.html>

17 March (0-1-12 8056)Over 90 percent of the major industrial enterprises in China's provincial-level regions except for certain areas including Hubei, once the hard-hit province of the novel coronavirus, have resumed work and production

<http://english.www.gov.cn/statecouncil/ministries/202003/17/content_WS5e7066b3c6d0c201c2cbe82c.html>

19 March (0-0-39 6569)Nearly half of major industrial enterprises in Central China's Hubei province have resumed production

<http://english.www.gov.cn/news/topnews/202003/19/content_WS5e72c23cc6d0c201c2cbea20.html>

About 96.4 percent of farm produce markets nationwide have resumed operation.

<http://english.www.gov.cn/statecouncil/ministries/202003/19/content_WS5e7379e3c6d0c201c2cbefad.html>

21 March (1-0-45 5549)Around 60 percent of medical services have been resumed in China amid the novel coronavirus disease (COVID-19) outbreak based on year-on-year comparison.

<http://english.www.gov.cn/statecouncil/ministries/202003/21/content_WS5e75a732c6d0c201c2cbf189.html>

The construction on 89.1 percent of 11,000 key projects (those in hard-hit Hubei province are not included) had restarted. In breakdown, 97.8 percent of the 533 key transportation projects supervised by the NDRC have been under construction, with all the major railway projects resuming operation, said Zheng Jian, another official with the NDRC. The construction on some 97 percent of major highway and waterway projects, 87 percent of airport projects, and 86 percent of water conservancy projects also resumed, said Ou.

<http://english.www.gov.cn/statecouncil/ministries/202003/21/content_WS5e76080ac6d0c201c2cbf1be.html>

24 March (0-0-47 4287)Delivery companies have gradually resumed normal operations in Wuhan, the city hit hardest by the novel coronavirus outbreak.

<http://english.www.gov.cn/news/topnews/202003/24/content_WS5e7963abc6d0c201c2cbf4b1.html>

25 March (0-0-67 3947) Construction has resumed on 65 airport projects across China, representing more than 80 percent of the country's airport projects under construction, according to the civil aviation authorities.

<http://english.www.gov.cn/statecouncil/ministries/202003/25/content_WS5e7b5582c6d0c201c2cbf74b.html>

27 March (0-0-54 3128)China's TV drama industry, which has been dormant for months due to the COVID-19 outbreak, is resuming work and production in an orderly manner, according to the National Radio and Television Administration

<http://english.www.gov.cn/statecouncil/ministries/202003/27/content_WS5e7de353c6d0c201c2cbf9fc.html>

30 March (0-0-48 2161)China's manufacturing sector has steadily advanced production resumption, with 98.6 percent of major industrial firms nationwide having restarted work Meanwhile, 76 percent of small and medium-sized enterprises have restarted to work nationwide.

<http://english.www.gov.cn/statecouncil/ministries/202003/30/content_WS5e818989c6d0c201c2cbfc4f.html>

6 April (0-0-32 1242)the work resumption rate of industrial enterprises above designated size in Wuhan, capital of Central China's Hubei province, has reached 97.2 percent by April 4, and 93.2 percent of leading service companies have resumed business.

<http://english.www.gov.cn/news/topnews/202004/06/content_WS5e8a6709c6d0c201c2cc03b4.html>

16 April (11-0-15 1081)Some 72.8 percent of key foreign-invested firms have recovered over 70 percent of work capacity as of April 14, up 0.9 percentage points compared with last week.

<http://english.www.gov.cn/news/topnews/202004/16/content_WS5e9869f5c6d0b3f0e949598e.html>

17 April (10-0-17 1058)work resumption rate for SMEs reached 84 percent

<http://english.www.gov.cn/news/topnews/202004/17/content_WS5e997768c6d0b3f0e9495be6.html>

20 April (7-0-4 1003)99.4 percent of central SOEs have resumed production, driving the upstream and downstream medium, small and micro-sized businesses to reboot engines in tandem, according to the State-owned Assets Supervision and Administration Commission of the State Council.

<http://english.www.gov.cn/news/topnews/202004/20/content_WS5e9d8d88c6d0b3f0e9495f82.html>

21 April (7-0-23 1005)China's agriculture-related firms have basically resumed normal operation, with over 98 percent of key agricultural material producers recovering production

<http://english.www.gov.cn/news/topnews/202004/21/content_WS5e9eb4efc6d0b3f0e94960be.html>

26 April (1-0-2 723)Central China's Hubei province, once hard hit by COVID-19, has resumed the operation of its 266 major Class-A tourist attractions, or 63.2 percent of the total, as the epidemic wanes, local authorities said on April 26.

<http://english.www.gov.cn/news/topnews/202004/26/content_WS5ea52012c6d0b3f0e9496662.html>
